# System Identification of Conveyor Belt Microwave Drying Process of Polymer Foams Using Electrical Capacitance Tomography

**DOI:** 10.3390/s21217170

**Published:** 2021-10-28

**Authors:** Marzieh Hosseini, Anna Kaasinen, Mahdi Aliyari Shoorehdeli, Guido Link, Timo Lähivaara, Marko Vauhkonen

**Affiliations:** 1 Department of Applied Physics, University of Eastern Finland, 70211 Kuopio, Finland; anna.kaasinen@uef.fi (A.K.); timo.lahivaara@uef.fi (T.L.); marko.vauhkonen@uef.fi (M.V.); 2 Mechatronics Department, Electrical Engineering Faculty, K.N. Toosi University of Technology, Tehran 16315-1355, Iran; aliyari@kntu.ac.ir; 3 Institute for Pulsed Power and Microwave Technology, Karlsruhe Institute of Technology, 76131 Karlsruhe, Germany; guido.link@kit.edu

**Keywords:** microwave drying, modeling, system identification, industrial tomography, electrical capacitance tomography

## Abstract

The microwave drying process has a wide application in industry, including drying polymer foams after the impregnation process for sealings in the construction industry. The objective of the drying process is to reach a certain moisture in the foam by adjusting the power levels of the microwave sources. A moisture controller can be designed to achieve this goal; however, a process model is required to design model-based controllers. Since complex physics governs the microwave drying process, system identification tools are employed in this paper to exploit the process input and output information and find a simplified yet accurate model of the process. The moisture content of the foam that is the process output is measured using a designed electrical capacitance tomography (ECT) sensor. The ECT sensor estimates the 2D permittivity distribution of moving foams, which correlates with the foam moisture. Experiments are conducted to collect the ECT measurements while giving different inputs to the microwave sources. A state-space model is estimated using one of the collected datasets and is validated using the other datasets. The comparison between the model response and the actual measurements shows that the model is accurate enough to design a controller for the microwave drying process.

## 1. Introduction

The microwave drying process is a promising technology for drying dielectric materials because of volumetric and selective heating, resulting in fast water evaporation [1,2]. Considerable energy and time efficiency are important features of this technology and are acquired by applying a high-power microwave (MW) to materials with high moisture content [3,4].

One of the main objectives of the microwave drying process is to reach a desired moisture content in the drying material after the process [3,5]. A moisture controller can be designed for the microwave oven to adjust the power level of the microwave sources accordingly. However, one of the requirements for developing a control system for this process is a mathematical model that simulates the process behavior with high accuracy.

The physics behind the microwave drying process is very complex as the equations governing the process depend on both time and position [6]. These types of systems are called distributed parameter systems (DPS), and they are typically modeled with partial differential equations (PDEs). Two coupled PDEs are required to model the heat and moisture transfer in the microwave drying process, making the modeling more challenging. Several studies have tried to apply the Luikov heat and moisture transfer model to a batch microwave drying process [7,8,9]. In [10,11], a pair of parabolic PDEs describing the heat and mass transfer were presented for the microwave drying of woods, and in Hosseini et al. [10], an LQR control method was designed for the simulated microwave drying process. However, a drawback of the proposed models is the unavailability of the exact model parameters for different materials. Furthermore, finding a dynamic model for the microwave drying process becomes more challenging when a conveyor belt system is studied instead of a batch process.

An alternative technique to avoid the complexity of the physics in modeling the process and overcome the unknown parameters is to employ system identification tools to model the process. System identification is a technique for obtaining a mathematical model of a system by processing the collected input and output data of the system [12,13,14]. System identification has been investigated in modeling several drying [15,16,17] and heating processes [18,19]. In Krishna Murthy and Manohar [15], the authors used experimental data from a microwave drying process with different power levels to choose the best thin-layer drying model among 15 candidates. The collected data were employed to estimate the chosen model parameters. In Li et al. [16], a recurrent self-evolving fuzzy neural network (RSEFNN) predictive control method was proposed for the microwave drying process. The advantage of this method was that the control algorithm employed the prediction of the microwave drying process behavior, and there was no longer a need for a mathematical model of the process.

Most research studies on the microwave drying process have studied a batch process, and the material moisture was rarely used as the system output. In Lutfy et al. [20], a conveyor belt electric dryer for paddy grains was modeled. In this paper, the moisture of the dried grains was used as the system output, while the speed of the conveyor belt was the corresponding input signal. However, the moisture measurement device in this study could only measure the moisture in a stationary state, so the authors could not run a continuous process. The input–output data were collected in several separate experiments, with the moisture measurements at the end of each experiment. The authors in Lähivaara et al. [21] studied microwave tomography to estimate the moisture distribution in the microwave drying process using the simulated data.

In this research, the microwave drying of polymer foams, a continuous process in the heat insulation industry, is studied. This process involves a polymer foam passing on a conveyor belt into a microwave oven equipped with several microwave sources. The power level of the microwave sources can be adjusted to control the moisture content of the polymer foams after the drying process. An electrical capacitance tomography (ECT) sensor was successfully developed in Hosseini et al. [22] to estimate the 2D permittivity distribution of polymer foams. Since the permittivity correlates strongly with moisture, the moisture of the foam can be estimated using a calibration map. The benefit of using the ECT sensor in this process is that it can estimate the moisture distribution of the material, unlike the common moisture measurement methods, where the point or average measurements are conducted. Furthermore, the material does not need to stay still during the measurement with the ECT sensor, and the moisture can be estimated while the material is moving on the conveyor belt.

The focus of the research presented in this paper is to estimate the polymer foam permittivity at different power levels of the microwave sources to model the microwave drying process. Furthermore, the effect of the input foam moisture (before entering the oven) on the output foam permittivity is also modeled. The first aim of this research is to find a single-input single-output (SISO) model of the process, and in future studies, a multi-input multi-output (MIMO) model will be derived.

Electrical capacitance tomography is a contactless, non-intrusive sensor that measures the electrical capacitances between electrodes mounted around the target material. A reconstruction algorithm is then used to estimate the permittivity distribution inside the imaging area. The efficiency of the ECT sensor in estimating the moisture content of materials in both stationary and dynamic measurements has been proven in several studies [22,23,24,25]. The ECT measurements are fast and can be employed for output signal collection in a continuous process. The process-specific requirements distinguish the ECT sensor used in this research from the conventional sensors. Since the foam has a rectangular cross-section and a large width, the non-neighboring electrodes on the ECT sensor are far apart, and as a result, the measured electrical capacitances between those electrodes are very low (less than 5 fF). However, the designed sensor has shown high enough accuracy in estimating the moisture content of the polymer foams in a microwave drying process [22].

Several experiments are designed in this study with different input signals for the power levels to collect the input foam moisture and the output foam permittivity. The collected data are employed to find a state-space model of the microwave drying process using the system identification tools.

## 2. Microwave Drying Process

**Figure 1:**
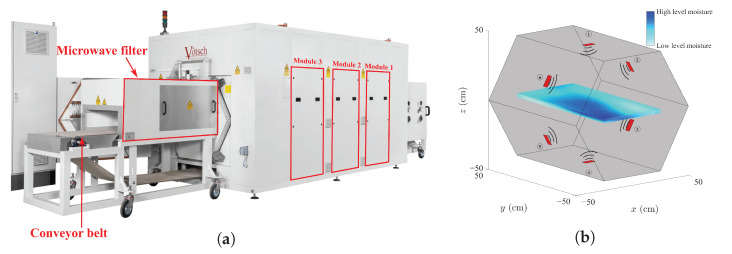
The microwave drying oven: (**a**) The HEPHAISTOS microwave system operating at KIT, Germany. (**b**) A schematic of one of the cavity modules with six microwave sources.

Microwave ovens can have different designs in terms of the number of microwave sources (magnetrons) installed, and the length and geometry of the oven. They also differ in terms of employing batch drying or continuous drying with a conveyor belt. This research studies a conveyor belt microwave oven named HEPHAISTOS, located at the Karlsruhe Institute of Technology (KIT), Germany. The HEPHAISTOS microwave oven is shown in Figure 1a. There are three cavity modules, each of 100 cm in length and equipped with six magnetrons operating at 2.45 GHz with 2 kW power [19]. The magnetrons are the sources of energy or the actuators in this system. Figure 1b illustrates a schematic of one of the modules and its hexagonal shape. Additionally, two microwave filters with a length of 150 cm are installed at the entrance and exit of the microwave system to prevent possible microwave leakage. The length of the whole microwave system is 729 cm. The HEPHAISTOS microwave oven is a combination oven with convective heating, meaning that the circulating hot air allows the transport of the evaporated water.

The test material in this study is a polymer foam with a density of $23\pm 2\;\mathrm{kg}/m^{3}$ that has application in the heat insulation industry. The microwave drying of polymer foams is a part of the impregnation process in which the polymer foams are first dipped into a bath of water and chemicals to form new features suited for sealing purposes. The wet foam then passes on a moving conveyor belt into the microwave oven, where 18 magnetrons provide volumetric heating, resulting in moisture evaporation from the foam. The process aims to keep the moisture level of the foam at a certain level and as homogenous as possible when it exits from the other side of the oven. The surface foam temperature can be monitored during the drying process using three IR cameras installed in the oven cavity modules. However, we cannot use the temperature data to estimate the internal moisture of the foam, and there is no information on the foam moisture throughout the process.

## 3. Materials and Methods

### 3.1. Electrical Capacitance Tomography

Electrical capacitance tomography (ECT) is a contactless, non-invasive measurement technology that can estimate dielectric material moisture distribution. In this research, the ECT sensor was installed at the exit of the oven and used to estimate the permittivity distribution of polymer foams after the drying process. Since the foam permittivity correlates with the foam moisture, a calibration map between the actual moisture values and the estimated permittivity can be used to obtain the foam moisture.

In general, the ECT sensor contains several electrodes installed on a structure around the target material. The measurement procedure starts by exciting one of the electrodes while the rest are electrically grounded. The capacitances between the exciting electrode and the other electrodes are measured, and the same procedure is repeated for all electrodes until one set of measurements is completed. With $N_{\mathrm{el}}$ electrodes, $m=N_{\mathrm{el}}\left(N_{\mathrm{el}}-1\right)/2$ measurements are collected at each sample time. Each round of measurements takes less than 1 s.

**Figure 2:**
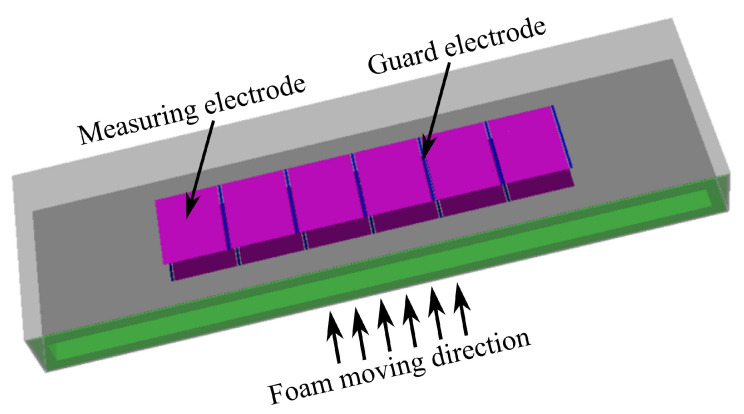
The ECT sensor design: six measuring electrodes and six grounded guard electrodes on the top surface and the same number of electrodes on the bottom surface.

Figure 2 shows the design of the ECT sensor developed for this research. Because of the small thickness of the foam (3 cm), no electrodes were installed on the sides of the sensor. Six electrodes were mounted on the top surface and six electrodes on the bottom surface of the sensor. Additionally, a thin, electrically grounded electrode was mounted between every adjacent electrode to increase the sensitivity of the measurements. However, these guard electrodes were not involved in the measurement procedure.

The polymer foam 2D cross-section had a rectangular domain with a width of 49.3 cm, which dictated a wide rectangular shape for the ECT sensor structure, while most developed ECT sensors are round [25,26,27]. This unusual structure imposed a considerable distance between non-neighboring electrodes, resulting in a weak measurement signal. Moreover, since the foam should easily pass through the sensor, a 1 cm air gap was maintained between the foam and the top surface electrodes. These practical limitations required a sensor design that minimized their effect on the measurement signal.

**Figure 3:**
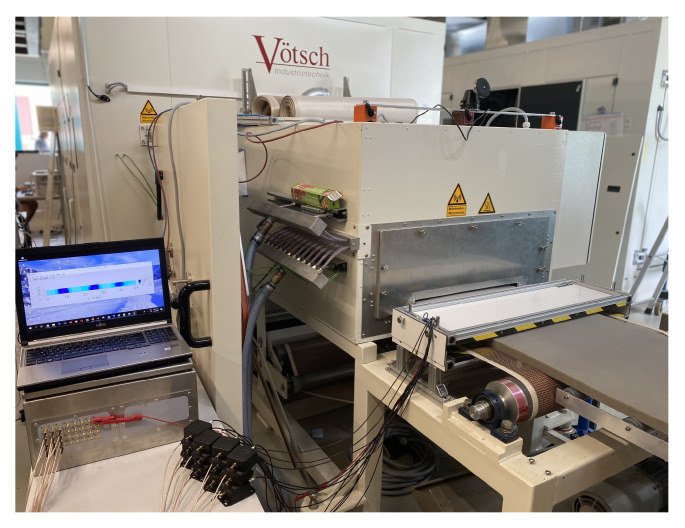
The ECT sensor installed at the exit of the microwave oven. After the drying process, the foam enters and passes through the ECT sensor while the ECT estimates its moisture distribution.

Several simulations and experiments with different numbers of electrodes and different electrode sizes resulted in our final design [22], as shown in Figure 2. The size of the whole sensor was 87cm×25cm×4cm with an adjustable height. The measurement electrodes were 10cm×8.1cm, and the guard electrodes were 10cm×0.3cm. The ECT sensor built according to the described design is shown in Figure 3, while it was installed at the exit of the microwave oven, estimating the foam permittivity after the drying process. The sensor was connected through cables to a measurement device manufactured by Rocsole Ltd., Kuopio, Finland. The role of the measurement device was to apply AC voltage to the ECT electrodes and measure the inter-electrode capacitances.

The measured electrical capacitances were employed in a reconstruction algorithm to estimate the permittivity distribution of the target material. The reconstruction algorithm in ECT includes solving both the forward problem and the inverse problem. The forward problem refers to a well-posed problem that connects the measured capacitances to the material permittivity. Our research uses the complete electrode model [26,28] to formulate the forward problem, and the equations corresponding to this model can be found in Hosseini et al. [22]. The finite element method is employed to solve the forward model numerically. By stacking the models for each measurement, the observation model for the ECT can be determined as

(1) C=H(ϵ)+v,

where $C={\left[C_{1},\ldots ,C_{m}\right]}^{T}$ is the vector of the measured inter-electrode capacitances, $\epsilon ={\left[\epsilon_{1},\ldots ,\epsilon_{n}\right]}^{T}$ denotes the discretized permittivity distribution, *n* is the number of the discretization points, $H\left(\epsilon \right)={\left[h_{1}\left(\epsilon \right),\ldots ,h_{m}\left(\epsilon \right)\right]}^{T}$ is the map between the permittivity distribution and capacitances, and the additive measurement noise is represented by $v={\left[v_{1},\ldots ,v_{m}\right]}^{T}$.

The inverse problem is an ill-posed problem that aims to determine the permittivity distribution with measured capacitances. There are different techniques used to solve the inverse problem [29,30,31,32]. These methods include linear and nonlinear methods as well as recursive and non-recursive methods. Furthermore, the accuracy and the computational time of the methods can vary. In this study, the difference imaging method is adopted to solve the inverse problem and to estimate the 2D permittivity distribution [26]. The real-time application and the need for a low computational time were the reasons for choosing the difference imaging method as it is a linear and computationally inexpensive method.

The first step in the difference imaging technique is to linearize the observation model (1) by the first-order Taylor polynomial. Two sets of measurements are collected in this method: the capacitance measurement while the dry material is placed inside the sensor, $C_{\mathrm{ref}}$, and the capacitance measurement with the wet foam, $C_{M}$. The approximated linear model can then be calculated using these two sets of measurements as

(2) $$\Delta C=C_{M}-C_{\mathrm{ref}}=J\Delta \epsilon +\Delta v,$$

where $\Delta \epsilon =\epsilon -\epsilon_{\mathrm{ref}}$ is the permittivity change between the wet and the dry material, $\epsilon_{\mathrm{ref}}$ is the dry foam permittivity, $J\left(\epsilon^{*}\right)=\partial H\left(\epsilon^{*}\right)/\partial \epsilon$ is the Jacobian matrix, and $\epsilon^{*}$ is the linearization point which is the best homogeneous (one value) estimate of the dry foam permittivity.

The inverse problem aims to calculate Δϵ by having the information on ΔC and solving an optimization problem. The final solution of the difference imaging technique can be stated as

(3) $$\Delta \epsilon =K\left(C_{M}-C_{\mathrm{ref}}\right),$$

where *K* is the reconstruction matrix calculated as [33]

(4) $$K={\left(J^{T}\Gamma_{\Delta v}^{-1}J+\Gamma_{\Delta \epsilon }^{-1}\right)}^{-1}J^{T}\Gamma_{\Delta v}^{-1},$$

where $\Gamma_{\Delta v}$ is the covariance matrix of the measurement noise term Δv and $\Gamma_{\Delta \epsilon }$ is the covariance matrix of the smoothness promoting prior. For a more detailed description, see [22] and references therein.

### 3.2. Input–Output Data Collection

The microwave drying process operated in the heat insulation industry is a continuous process, meaning that a very long polymer foam sheet passes first through the impregnation bath and then enters into the microwave oven. However, we did not have a long foam available to simulate a continuous process, as used in the industry. Instead, several polymer foam sheets with a length of 150 cm were available. These foams had a thickness of 3 cm and a width of 49.3 cm. The experiments aimed to use these sheets right after each other to form a quasi-continuous foam.

**Figure 4:**
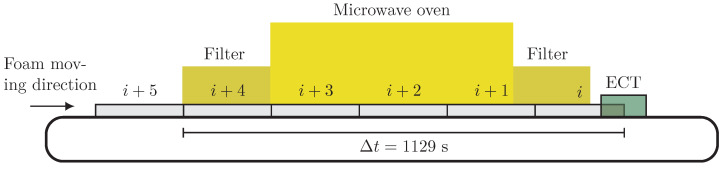
The schematic of the continuous drying process of polymer foams. The gray rectangles indicate the polymer foams with a length of 150 cm and thickness of 3 cm while passing first through the oven and then the ECT sensor with a speed of 40 cm/min. It took 1129 s for every foam to travel from the entrance point and reach the ECT sensor.

Figure 4 shows a schematic of the foam sequence entering the oven. At any time, there were five foams between the entrance point and the center of the ECT sensor. The conveyor belt speed was 40 cm/min, meaning that it took 1129 s for any point of the foam to travel from the entrance of the microwave oven to the middle of the ECT sensor. This way, when the foam sheet *i* reached the middle of the ECT sensor, the foam sheet i+5 entered the oven.

In this research, the system identification aims to find a dynamic model for the microwave drying process that connects the system inputs to the system outputs. Input–output data collection is the first step of system identification. Section 3.3 and Section 3.4 specify the inputs and outputs of the system and their recording procedure. The sample time in input–output data collection was selected as 1 s, which is an appropriate value considering the slow belt speed.

### 3.3. System Inputs

The sources of heating energy in the microwave drying process were 18 installed magnetrons. The microwave system was operated by software named SIMPAC developed by the manufacturing company Weiss Technik GmbH, Germany. The power percentage of the magnetrons, among some other parameters, can be adjusted using SIMPAC. Moreover, there was an interface developed at KIT that provided the possibility of interacting with SIMPAC through MATLAB.

The magnetrons had a maximum power of 2 kW and could be activated by giving their input a percentage level between 0% and 100%. However, any power lower than 15% was the same as 0% since magnetrons need a minimum power of about 15% to become activated. Thus, it was recommended to choose a minimum of a 15% power level as the inputs to the magnetrons. Since this research aimed to obtain a SISO model, the same power level ($P_{l}=P$, l=1,…,18) was given to all magnetrons at all times in the following experiments. The power percentage of the magnetrons, *P*, was taken as the first system input ($u_{1}=P$).

**Figure 5:**
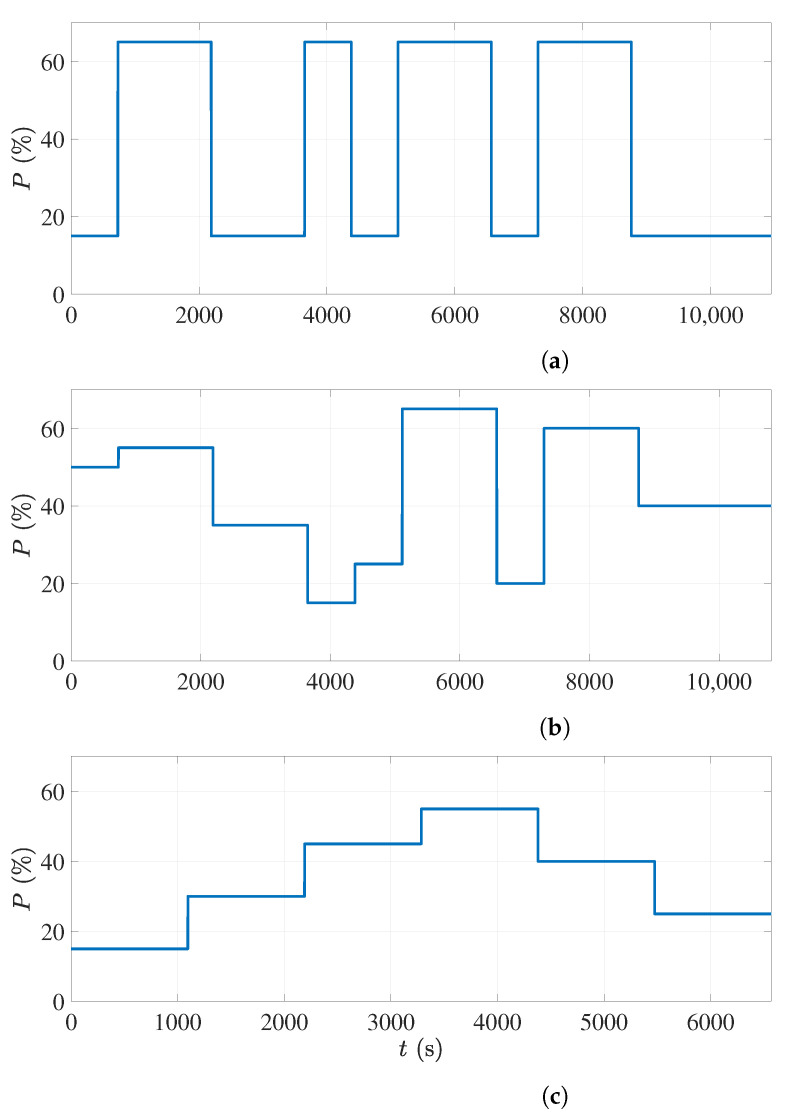
Input signals for the system identification: (**a**) PRBS signal. (**b**) APRBS signal. (**c**) Step signal as staircase.

Choosing an appropriate input signal to model a process is very important [34]. In this research, three different standard signals were selected to be applied as the power percentage of the microwave sources [35]. The first signal was a pseudorandom binary sequence (PRBS) signal, the second signal was an amplitude-modulated pseudorandom binary sequence (APRBS) signal, and the third was a step signal with increasing and decreasing levels (staircase). Figure 5 shows all the three input signals used as the power percentage of all microwave sources in the input–output data collection. The pulse width, the minimum, and the maximum power level in these signals were chosen based on the process time delay and the sample time.

In addition to the power level of microwave sources, the input foam moisture also affected the system output. Although the ideal scenario was to keep the input foam moisture as constant as possible, there was still a variation in the input foam moisture. The unwanted change in the input foam moisture was taken as the second input to the process ($u_{2}=M_{\mathrm{in}}$) to model its effect on the system output. The sheets of wet foams were weighed on a digital scale before passing through the microwave oven, and their moisture content on a wet basis was calculated as

(5) $$M_{\mathrm{in}}=\frac{W_{w}-W_{d}}{W_{w}}\times 100,$$

where $W_{d}$ is the weight of the dry foam and $W_{w}$ is the weight of the wet foam. Since only the average moisture was available for each foam sheet, the moisture content throughout the foam length, equivalent to 225 s of travel time on the conveyor belt, was considered constant.

### 3.4. System Outputs

The system output is the moisture of the polymer foam during the drying process. The designed ECT sensor could not be installed inside the microwave oven due to safety issues, and it was located right after the microwave exit, as shown in Figure 3. Therefore, the moisture information of the material was only available after the drying process.

As explained in Section 3.1, the ECT sensor estimates the permittivity change of the foam, and the material moisture can be calculated using a calibration map. However, to avoid unnecessary calibration errors, it was decided to take the material permittivity change directly as the system output. Furthermore, the ECT estimates the 2D permittivity distribution of the target polymer foam. For a SISO model, it was sufficient to use only the average permittivity change of the material. When obtaining a MIMO model, the permittivity distribution will be utilized. As in this research the material moisture is studied on a wet basis, the average permittivity change calculated from (3) can be converted to the permittivity change in wet basis, $\Delta \epsilon_{w}$, by

(6) $$\begin{array}{cc} \Delta \epsilon_{d} & =\frac{\bar{\Delta \epsilon }}{\epsilon_{\mathrm{ref}}}\times 100, \end{array}$$

(7) $$\begin{array}{cc} \Delta \epsilon_{w} & =\frac{\Delta \epsilon_{d}}{100+\Delta \epsilon_{d}}\times 100, \end{array}$$

where $\bar{\Delta \epsilon }$ is the average value of Δϵ at the *n* nodes in the 2D coordinate, and $\Delta \epsilon_{d}$ is the dry basis permittivity change (in percentage).

### 3.5. Analyzing the Collected Data and Identifying the Process Model

**Figure 6:**
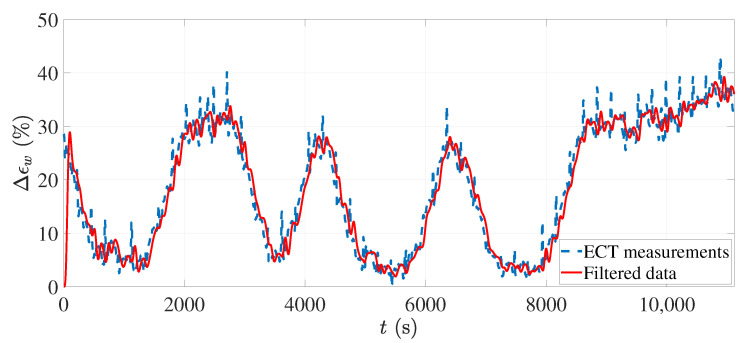
The collected ECT sensor measurements before and after employing a stopband filter.

**Figure 7:**
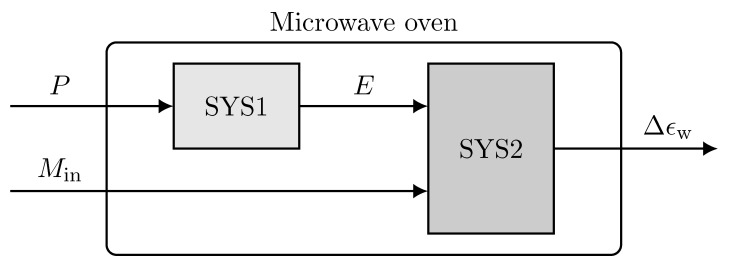
Dividing the microwave oven system into two subsystems.

Collecting the input–output dataset was the first step in the system identification process. The collected data often need to be preprocessed before using the dataset in the system identification algorithms. Some preprocessing methods are standard procedures that are conducted regardless of the system. However, knowing the physics and principles of the process can help to choose the suitable preprocessing techniques for that specific process. In this research, the following preprocessing procedure was conducted on the collected datasets:

1. The data collected from any sensor are usually accompanied by unwanted measurement noise, which can be resolved by filtering the data. The measurement noise was trivial with the ECT sensor because of the efficient design and the reconstruction algorithm. However, since we used foam sheets instead of a very long continuous foam, the measurements had high peaks corresponding to the edge of foams. As observed during the experiments, drops of water were usually accumulated on the edges, resulting in increased moisture recognition. A stopband filter was applied to the collected dataset to remove high-frequency measurements. Figure 6 shows the collected and filtered output data.
2. The objective of this research was to find a linear model of the process. Linear models cannot catch arbitrary differences between the input and output signal levels. Therefore, the mean values were removed from the input–output data. Removing the mean values (constant term) allows the analysis of the other signal contents, resulting in a more accurate model.
3. Since the ECT sensor was located after the process, it was not straightforward to find the relation of the input power at each time to the measured output. Any point of the foam traveling through the ECT sensor was exposed to several power levels while traveling inside the oven. Therefore, in this study, the system was divided into two subsystems, SYS1 and SYS2, by introducing a virtual input, as shown in Figure 7. The new input signal, *E*, is the overall applied energy to each location of the foam that was calculated by integrating the power level signal in the travel time corresponding to that location as
  (8) $$E\left(t\right)=\int_{t-t_{f}-t_{c}}^{t-t_{f}}P\left(t\right)dt,$$
  where $t_{c}$ is the travel time inside the oven cavity (three modules) and $t_{f}$ is the travel time inside the microwave filter. The travel time inside the microwave filter is excluded in (8) since there is no significant power applied to the foam inside the microwave filter. The SYS1 is the model between the input power level, *P*, and the overall applied energy, *E*, and the SYS2 models the new input, *E*, and the input moisture, $M_{\mathrm{in}}$, to the system output, $\Delta \epsilon_{w}$.

The preprocessed data were used to find a linear model that fit best for SYS1 and SYS2. The model representing the microwave oven could then be achieved by connecting these two models. The MATLAB system identification toolbox 9.10 was employed to fit a linear model. Different linear models were tested for both SYS1 and SYS2.

Considering the relation between P(t) and E(t) in (8), SYS1 is an integrator over a specific time interval, so a transfer function with time delay was a reasonable choice for this system. Different transfer function models with different numbers of zeros and poles were tested to find the best model comparing their goodness of fit. Eventually, a discrete-time transfer function with two poles, one zero, and an input time delay was determined for SYS1. The identified model can be stated as

(9) $$\frac{E(z)}{P(z)}=z^{-T_{d1}}\frac{bz^{-1}}{1-a_{1}z^{-1}+a_{2}z^{-2}},$$

where E(z) and P(z) are the Z-transforms of the discrete-time signals E[k] and P[k], respectively, *k* represents the sample number, and the input time delay is denoted by $T_{d1}$. The unknown model parameters *b*, $a_{1}$, $a_{2}$, and $T_{d1}$ were calculated using the collected values for E[k] and P[k].

The SYS2 was modeled with a state-space model as most of the intended control algorithms employ a state-space model of the process. The order of the state vector in this model (the system order) was determined automatically by the system identification toolbox given the input and output signals, such that it gave the best goodness of fit with the lowest system order. The identified model has six states, two input signals, and one system output. The first input, *E*, has no time delay as it was defined by us, while the second input, $M_{\mathrm{in}}$, has a time delay of $T_{d2}$ since it was measured before passing the foam into the oven. The equations describing this model are

(10) $$\begin{array}{cc} \; & x\left[k+1\right]=Ax\left[k\right]+B_{1}E\left[k\right]+B_{2}M_{\mathrm{in}}\left[k-T_{d2}\right], \end{array}$$

(11) $$\begin{array}{cc} \; & y[k]=Cx[k], \end{array}$$

where $x\left[k\right]\in R^{6\times 1}$ is the state vector, and the model output y(k)∈R is the model response for the estimated permittivity change percentage, $\Delta \epsilon_{w}$. The matrices $A\in R^{6\times 6}$, $B_{1},B_{2}\in R^{6\times 1}$, and $C\in R^{1\times 6}$ are the model parameters estimated using the system identification toolbox given the collected inputs and output data.

The augmented model of the system can be obtained by connecting SYS1 and SYS2, resulting in a state-space model as

(12) $$\begin{array}{cc} \; & X\left[k+1\right]=A_{\mathrm{aug}}X\left[k\right]+B_{1,\mathrm{aug}}u_{1}\left[k-T_{d1}\right]+B_{2,\mathrm{aug}}u_{2}\left[k-T_{d2}\right], \end{array}$$

(13) $$\begin{array}{cc} \; & y\left[k\right]=C_{\mathrm{aug}}X\left[k\right], \end{array}$$

where $X\left[k\right]\in R^{8\times 1}$ is the state vector of the whole system with eight elements resulting from the connection of a state-space model with the order of six stated in (10) and (11) to the transfer function with the degree of two in (9). The input signals are the input power level to the microwave sources, $u_{1}=P$, with $T_{d1}$ time delay for samples, and the input foam moisture, $u_{2}=M_{\mathrm{in}}$, with $T_{d2}$ time delay for samples. The matrices $A_{aug}$, $B_{1,\mathrm{aug}}$, $B_{2,\mathrm{aug}}$, and $C_{\mathrm{aug}}$, which are the model parameters for the augmented system, can be easily determined by connecting the state-space model (10) and (11) to the transfer function (9) in MATLAB software using the connect command. The sample time for all the represented models in this section was 1 s.

## 4. Results and Discussion

Different datasets with the input signals shown in Figure 5 were collected while running the continuous microwave drying of polymer foams. All the recorded inputs and outputs were preprocessed as described in Section 3.5. In this research, the dataset with the PRBS input signal was used to estimate the unknown parameters of the transfer function (9) and the state-space model (10) and (11).

The model parameters in (9), including the input time delay, $T_{d1}$, were estimated using the MATLAB system identification toolbox, employing the least square methods. The estimated parameters are given in Table 1. The matrices *A*, $B_{1}$, $B_{2}$, and *C* in the state-space model (10) and (11) were estimated using the subspace method [36] and are

(14) $$A=\left[\begin{array}{cccccc} 0.9987 & -0.0231 & -0.0093 & 0.0048 & -0.0077 & -9.623\times 10^{-5}\\ 0.0228 & 0.9984 & 0.0507 & 0.0011 & -0.0001 & 1.269\times 10^{-6}\\ 0.0098 & -0.0499 & 0.997 & -0.0409 & -0.0020 & 0.0002\\ -0.0047 & -0.0035 & 0.0416 & 0.9963 & 0.0753 & 9.458\times 10^{-5}\\ 0.0072 & 0.0001 & -0.0001 & -0.0754 & 0.9919 & -0.131\\ 8.531\times 10^{-5} & 0.0008 & 0.0008 & -0.0038 & 0.0887 & 0.8314 \end{array}\right],$$

and

(15) $$B_{1}=\left[\begin{array}{c} -0.0227\\ -0.0160\\ 0.0841\\ 0.1604\\ -0.0316\\ -0.0077 \end{array}\right]\times 10^{-6},\;B_{2}=\left[\begin{array}{c} 0.0015\\ 0.0021\\ -0.0070\\ -0.0095\\ -0.0021\\ -0.0012 \end{array}\right],\;C={\left[\begin{array}{c} -154.440\\ -13.853\\ -16.738\\ -16.272\\ 0.070\\ 0.001 \end{array}\right]}^{T}.$$

Furthermore, the input time delay for the input moisture, $T_{d2}$, was measured as 1129 s, which is the foam’s travel time from the oven entrance to the ECT sensor.

The identified transfer function and the state-space model were connected to form the augmented model (12) and (13). The accuracy of the augmented model can be evaluated by applying the same inputs given to the actual microwave oven and then comparing the model response with the ECT measurements. The cost function used in this study to show the accuracy of the estimated system output is the normalized mean squared error (NMSE), which is calculated as

(16) $$\mathrm{fit}=\frac{{∥\Delta \epsilon_{w}-y∥}^{2}}{{∥\Delta \epsilon_{w}-\bar{\Delta \epsilon_{w}}∥}^{2}}\times 100,$$

where $\Delta \epsilon_{w}$ is the permittivity change percentage measured by the ECT sensor (the process output), $\bar{\Delta \epsilon_{w}}$ is the average value of this variable over time, and *y* is the identified model output from (13) given the same input signals.

**Figure 8:**
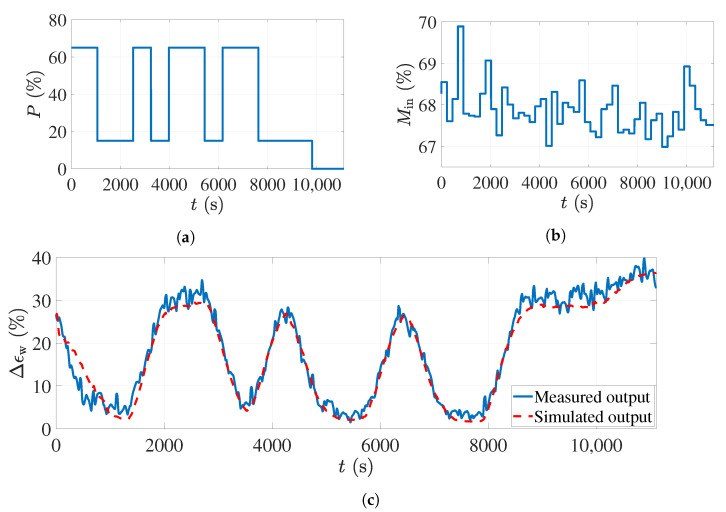
The input–output dataset with the PRBS input signal used for estimating the process model: (**a**) The applied input power percentage to the microwave sources. (**b**) The input foam moisture variations. (**c**) Comparison between the actual measurements and the model output.

The simulation and experimental results with the PRBS dataset are shown in Figure 8. The top-left subfigure is the applied power level percentage to the magnetrons, which is the PRBS signal. The minimum level in the PRBS signal was chosen to be 15% of the maximum power of the magnetrons as the magnetrons do not start working with less power than that. The maximum power percentage in this figure is 65%, since the risk of over-drying and burning the foam will increase with more power. The PRBS signal was programmed in MATLAB software and given to all magnetrons simultaneously through an interface connected to the oven.

The foam sheets were moisturized in the impregnation tub, containing only water, and weighed afterward using a digital scale to calculate their actual moisture before sending them to the oven (see Figure 4). One average moisture percentage on a wet basis was calculated for each foam sheet. Figure 8a shows the moisture percentages of the foams before entering the oven in this experiment. Since the foam sheet had a length of 150 cm and the conveyor belt was moving with a speed of 40 cm/min, the calculated average moisture value was constant at intervals of 225 s in Figure 8b.

While giving the PRBS signal to the magnetrons, the corresponding process output (foam moisture after the drying process) was calculated based on the ECT sensor measurements. The experimental measurements are shown in Figure 8c with a solid blue line. As mentioned, these data were used to estimate the parameters of the state-space model (12) and (13). The same inputs as shown in Figure 8a,b were applied to the derived model to reproduce the process output. The simulated process output is shown with the dashed red line in Figure 8c. As can be seen, the model output nicely follows the actual measurements. The time delay for the second input of the model (input foam moisture) was 1129 s, and this is why, in the first 1129 s of the simulation in Figure 8c, the model output does not fit the actual measurements well; however, after this interval, the tracking error decreases. The fit value calculated from (16) and using these data was 96.17%, which is very accurate.

The previous simulation showed that the derived model can reproduce the actual process measurements very accurately. However, since the same data were used to estimate the model parameters, the model performance should also be validated with other datasets called validation data. Two additional datasets with the APRBS and staircase signals as the input signal for the power levels were used as validation datasets.

**Figure 9:**
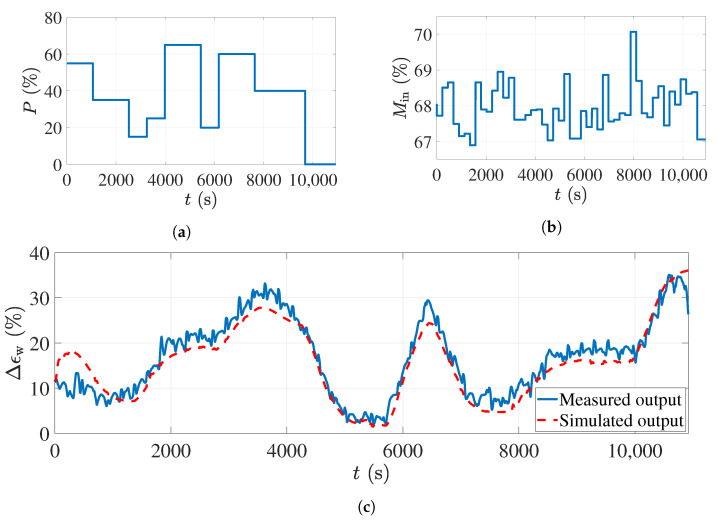
The input–output dataset with the APRBS input signal used for validation of the process model: (**a**) The applied input power percentage to the microwave sources. (**b**) The input foam moisture variations. (**c**) Comparison between the actual measurements and the model output.

Figure 9a shows the APRBS signal applied to the magnetrons in the second experiment. As with the PRBS signal, the minimum and maximum power levels in this signal were chosen as 15% and 65%, respectively. The difference from the previous experiment is that the power level could vary randomly between these limits during the experiment. The moisture percentages of the wet foams before entering the oven in this experiment are shown in Figure 9b

While the APRBS signal was given to the magnetrons, the ECT sensor measurements were collected, as shown in Figure 9c with a solid blue line. The simulated model output for this experiment is shown in red dashed line in the same figure. As can be seen, the process output with the APRBS signal showed a different pattern compared to the previous experiment with the PRBS signal. Nonetheless, the derived model was still able to estimate the process output with reasonable accuracy. The fit value for this data set was calculated as 87.8%, which is a great fit for a validation dataset. It should be noted that since the second model input had a time delay of 1129 s, the first 1129 s in the model verification showed a higher error compared to the rest of the data. Omitting the first 1129 s of the model response to evaluate the fit value increases the accuracy to 90.7%.

**Figure 10:**
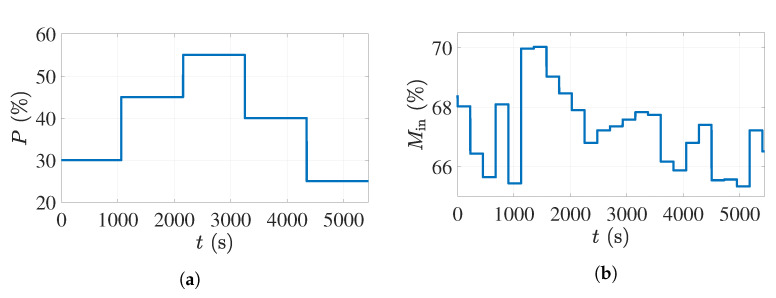

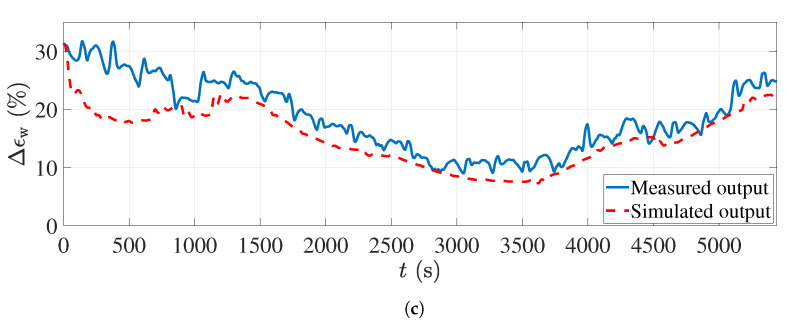
The input–output dataset with the staircase input signal used for validation of the process model: (**a**) The applied input power percentage to the microwave sources. (**b**) The input foam moisture variations. (**c**) Comparison between the actual measurements and the model output.

The second validation dataset was acquired when the staircase signal shown in Figure 10a was applied as the input to the magnetrons. The recorded moisture percentages of the foam sheets before the drying process in this experiment are shown in Figure 10b. Figure 10c illustrates the comparison between the estimated permittivity change and the simulated model output in this experiment. As can be seen, only for the first period of 1129 s, which is the second input time delay, is there a large error, and after that, the model showed a similar response to the sensor measurements. The accuracy of the model response can be calculated as 68.7% after omitting the first 1129 s from the model response.

## 5. Conclusions

In this paper, the system identification of a conveyor belt microwave drying process was investigated. The test material was polymer foam with sealing applications in the construction industry. An ECT sensor was installed at the exit of a microwave oven to estimate the polymer foam permittivity, which correlates with the foam moisture. The prespecified input signal for this process was the power level of the microwave sources, and the additional recorded input was the input foam moisture. In several experiments with different input power level signals, the corresponding output foam permittivity was estimated. The system identification methods were applied to the collected input and output signals, and a state-space model of the process was estimated. The accuracy of the obtained model was validated with different datasets. The estimated linear model can be employed with good accuracy for the design of a moisture controller for the microwave drying process with various linear control methods. Furthermore, the designed ECT sensor can estimate the moisture distribution of the material, and is thus suitable for deriving a MIMO model of the process. 

## Figures and Tables

**Table 1:** The parameters of the transfer function SYS1.

Parameter	*b*	$a_{1}$	$a_{2}$	$T_{d1}$
**Value**	5.109	−1.992	0.992	261

## Data Availability

The data presented in this study are openly available in Zenodo at 10.5281/zenodo.5509231.
